# Breast cancer resistance protein (Bcrp/*Abcg2*) is selectively modulated by lipopolysaccharide (LPS) in the mouse yolk sac

**DOI:** 10.1016/j.reprotox.2020.09.001

**Published:** 2020-12

**Authors:** L.M. Martinelli, M.W. Reginatto, K.N. Fontes, C.B.V. Andrade, V.R.S. Monteiro, H.R. Gomes, F.R.C.L. Almeida, F.F. Bloise, S.G. Matthews, T.M. Ortiga-Carvalho, E. Bloise

**Affiliations:** a Department of Morphology, Institute of Biological Sciences, Federal University of Minas Gerais, Brazil; b Laboratory of Translational Endocrinology, Institute of Biophysics Carlos Chagas Filho, Federal University of Rio de Janeiro, Brazil; c Departments of Physiology,Obstetrics and Gynecology and Medicine, University of Toronto, Canada; d Lunenfeld-Tanenbaum Research Institute, Mount Sinai Hospital, Ontario, Canada

**Keywords:** Prenatal endotoxemia, Lipopolysaccharide (LPS), Bacterial infection, Mouse yolk Sac, Abca1, Abcg1, Breast cancer resistance protein (Bcrp/*Abcg2*), P-glycoprotein (P-gp/*Abcb1a* and *Abcb1b*)

## Abstract

Bacterial infection alters placental ABC transporters expression. These transporters provide fetal protection against circulating xenobiotics and environmental toxins present in maternal blood. We hypothesized that lipopolysaccharide (LPS-bacterial mimic) alters the yolk sac morphology and expression of key ABC transporters in a gestational-age dependent manner. Yolk sac samples from C57BL/6 mice were obtained at gestational ages (GD) 15.5 and GD18.5, 4 or 24 h after LPS exposure (150ug/kg; n = 8/group). Samples underwent morphometrical, qPCR and immunohistochemistry analysis. The volumetric proportions of the histological components of the yolk sac did not change in response to LPS. LPS increased *Abcg2* expression at GD15.5, after 4 h of treatment (p < 0.05). No changes in *Abca1, Abcb1a/b, Abcg1, Glut1, Snat1, Il-1β, Ccl2* and *Mif* were observed. *Il-6* and *Cxcl1* were undetectable in the yolk sac throughout pregnancy. Abca1, breast cancer resistance protein (Bcrp, encoded by *Abcg2*) and P-glycoprotein (P-gp/ *Abcb1a/b)* were localized in the endodermal (uterine-facing) epithelium and to a lesser extent in the mesothelium (amnion-facing), whereas Abca1 was also localized to the endothelium of the yolk sac blood vessels. LPS increased the labeling area and intensity of Bcrp in the yolk sac’s mesothelial cells at GD15.5 (4 h), whereas at GD18.5, the area of Bcrp labeling in the mesothelium (4 and 24 h) was decreased (p < 0.05). Bacterial infection has the potential to change yolk sac barrier function by affecting Bcrp and *Abcg2* expression in a gestational-age dependent-manner. These changes may alter fetal exposure to xenobiotics and toxic substances present in the maternal circulation and in the uterine cavity.

## Highlights

• ABC transporters modulate transfer of xenobiotics, toxins, nutrients and cytokines.
• Bcrp is localized in the yolk sac endodermal epithelium and in the mesothelium.
• Lipopolysaccharide affect yolk Bcrp/*Abcg2* expression in a gestational-age dependent-manner.
• These changes may alter fetal exposure to xenobiotics and toxic substances.

## 1 Introduction

While controversial, there is some evidence of bacterial microbiomes in the uteri of healthy pregnancies [[1], [2], [3]]. Under pathological conditions, the intrauterine environment may be seeded with dysbiotic bacteria; originating from hematogenous transplacental passage, from ascending vaginal infection or from the peritoneal cavity (through the myometrium and into the trophoblast) [4,5]. As a consequence, infection and inflammation of the amnion and the chorion, as well as infection of the amniotic fluid, may take place and be referred to as chorioamnionitis [4]. Infective chorioamnionitis is amongst the most important pregnancy complications increasing the risk of preterm labor (PTL) and postnatal brain abnormalities [6,7]. Chorioamnionitis can be triggered by infective gram-negative bacteria [8], though it can also be triggered by other etiologies, such as gram-positive bacteria, viral and malarial infections [[8], [9], [10]]. Additionally, gram-negative bacteria infection in early pregnancy is associated with high rates of pregnancy loss and miscarriage [11], highlighting the need for a better understanding of the effect of gram-negative bacteria infection in intrauterine tissues through pregnancy.

The human yolk sac is a temporary membrane attached to the developing conceptus. It is involved in early hematopoiesis and formation of primordial germ cells. Emerging evidence has demonstrated that it also exerts vital barrier functions, allowing maternal-embryo nutrient transport exchange between the exocoelomic fluid and the developing embryonic structures. This categorizes the yolk sac as a transient nutritive organ [[12], [13], [14]]. In mice, the visceral portion of the yolk sac wraps the entire embryo and is functional throughout pregnancy, mediating the transport of critical substances from the mother to fetus [13,15]. In both species, the yolk sac expresses mRNA for the different classes of transmembrane transporter systems, including those belonging to the ATP-binding cassette (ABC) system of efflux transporters [14]. These transporter proteins are also expressed in placenta [19,45].

ABC transporters are transmembrane proteins responsible for actively transporting different substrates from the intra- to the extra-cellular environment. ABC transporters are expressed at high levels at tissue barrier sites, where they modulate transfer of an array of compounds involved in nutrition (lipids, amino acids, folate), immune response (pro-inflammatory cytokines and chemokines) and endocrine function (glucocorticoids, estrogens and progestins) [[16], [17], [18]]. In addition, ABC transporters can efflux environmental toxins and clinically relevant drugs out of cells, playing a key role in the biodistribution of xenobiotics and toxins.

Placental breast cancer related protein (BCRP, encoded by *ABCG2)* and P-glycoprotein (P-gp, encoded by *ABCB1* in humans and by *Abcb1a* and *Abcb1b* in rodents) are localized in the apical membrane of human syncytiotrophoblasts and mediate fetal protection against xenobiotics and environmental toxins that may be present in the maternal circulation. Substrates are effluxed from within the syncytiotrophoblast back towards the maternal circulation, thus decreasing their accumulation in the fetal circulation. In turn, ABCA1 effluxes cholesterol and cytotoxic oxysterols in the placental barrier towards the maternal side [19]. Of importance, infection and inflammation are potent modulators of placental multidrug resistance and ABC-lipid transporters expression and function [10,[19], [20], [21], [22]], demonstrating that infective challenges alter the fetal accumulation of nutrient, cytokines, hormones, drugs, environmental toxins and cytotoxic oxysterols, which may severely impact pregnancy outcome.

In mice, limited information about how bacterial infection impacts the yolk sac morphology and efflux transport potential is available. Studies on the expression of transporter proteins in mouse placenta challenged by LPS are in publication elsewhere [32], thus, we hypothesized that prenatal endotoxemia induced by lipopolysaccharide (LPS – modeling bacterial infection) alters the yolk sac morphology and expression of key ABC transporters in a gestational-age dependent manner. We investigated the tissue morphology and expression profile of important ABC transporters in the mouse yolk sac following LPS exposure, in order to better understand how bacterial agents impact yolk sac barrier efficiency. This new knowledge may expand the understanding of the mechanisms by which bacterial infection impacts the biodistribution of nutrients, immunological factors, hormones, drugs and toxins within the developing conceptus.

## 2 Material and methods

### 2.1 Animals

Virgin C57BL/6 J female mice (8–10 weeks) were time-mated with C57BL/6 J males and randomly sorted the next morning (gestational day (GD) 0.5) in groups timely exposed to LPS (from *Escherichia coli* 055:B5. Source/strain: CDC 1644-70. Lot number L2880; dose 150 ug/kg; i.p.) in different gestational ages. Pregnant females were separated into 4 groups: 1) treated with LPS (n = 8) or its vehicle (saline; n = 8) at GD15.5 and sacrificed 4 h after treatment; 2) treated with LPS (n = 8) or its vehicle (n = 8) at GD18.5 and sacrificed 4 h after treatment; 3) treated with LPS (n = 8) or its vehicle (n = 8) at GD14.5 and sacrificed 24 h after treatment; and 4) treated with LPS (n = 8) or its vehicle (n = 8) at GD17.5 and sacrificed 24 h after treatment. Euthanasia was performed with pentobarbitol overdose (300 mg/kg i.p.) and maternal/fetal decapitation. The LPS dose regimen of 150 ug/kg was selected based on its sublethal effects, i.e., it has been shown to induce severe maternal and placental inflammatory response associated with fetal death (less than 50 %) [21]. LPS treatment for 24 h at GD15.5, yielded a 87 % rate of fetal death which prevented us from undertaking further analysis in this time point, however, LPS induced 36 2 and 2 % rates of fetal death at GDs 15.5 (4 h), 18.5 (4 h) and 17.5 (24 h) respectively [32]. These groups were therefore, included in the present study. Institutional ethics committee approval (CEUA-190/13) was obtained and registered within the Brazilian National Council for Animal Experimentation Control (protocol number 01200.001568/2013-8). All procedures followed the “Principles of Laboratory Animal Care” advised by the National Society for Medical Research and the U.S. National Academy of Sciences Guide for the Care and Use of Laboratory Animals.

### 2.2 Mouse yolk sac morphometry

The volumetric proportion of the mouse visceral yolk sac from LPS-treated and control animals (n = 8/group) was assessed as previously described with modifications [23,24]. Briefly, after dissection from the amnion membrane, yolk sac specimens were included in buffered paraformaldehyde phosphate solution (4 %) for 24 h. Samples were then immersed in ethanol and xylene series and embedded in paraffin (Histopar, SP, Brazil). Hematoxylin and eosin (H&E) staining was performed in sections (4 μm) prior to image acquisition and analysis, which were undertaken on a Zeiss Axiolab 1 photomicroscope, coupled with a CCD camera and computer running Zeiss Axiovision (Carl Zeiss, NY, EUA). The morphometric analysis was performed using the Fiji ImageJ 1.0 program (ImageJ, WI, USA). Relative volume estimates of the yolk sac histological constituents (endodermic epithelium, mesodermal connective tissue, mesodermal blood vessels and mesothelium) were performed by superimposing histological photomicrographs with a grid of equidistant points (measuring 25 μm distance between two points). 1000 points overlapping with each of the yolk sac histological constituents were recorded, yielding a variable number of histological sections evaluated per dam; which corresponded to a total average area of 455 mm^2^ per dam. The volumetric proportion (VP) of each histological component was calculated as VP = NP × 100/1000, where NP = number of equivalent points on each histological component [24].

### 2.3 Immunohistochemistry

Yolk sac samples were processed for immunohistochemical analysis as previously described [22,23]. Mouse placental sections (GD18.5) were concomitantly processed, as positive controls. Endogenous peroxidase activity was blocked using the Hydrogen Peroxide Block kit (Springer, Berlin, Germany), whereas antigen retrieval was undertaken by microwave heating in citrate buffer (0.1 M, pH 6) for 10 min. Blocking of non-specific sites was performed by incubating the tissue slides with 10 % skimmed milk in PBS (30 min), followed by Protein Block kit (Springer; 30 min). Overnight incubation with anti-P-gp (mouse monoclonal-Mdr1[sc-55510]; Santa Cruz Biotechnology, EUA; 1:500), anti-Abca1 (mouse monoclonal-[ab18180]; Abcam, EUA; 1:100), anti-Bcrp (mouse monoclonal-[MAB4146]; Merck Millipore, GER; 1:200) primary antibodies was followed by incubation with biotin-conjugated secondary antibody (SPD-060 - Spring Bioscience, California, USA) for 1 h and streptavidin (SPD-060 - Spring Bioscience, California, USA) for 1 h. Chromogenic detection was achieved with 3,3-diamino-benzidine (DAB) (SPD-060 - Spring Bioscience, California, USA) exposure. Sections were stained with hematoxylin. Replacement of primary antibodies with normal serum provided negative controls.

Quantification of intensity and area of immunolabeling in yolk sac components, endodermal epithelium, connective tissue, endothelium and mesothelium, were performed using a semi-quantitative scoring described previously [22,25], with modifications [22,23]. Immunolabeled area was scored as follows: 0) undetectable; 1) 1–25 %; 2) 26–50 %; 3) 51–75 %; and 4) 76–100 %. Whereas, for intensity immunolabeling, graded scores were: 0) no detectable staining; 1) weak; 2) moderate; 3) strong; and 4) very strong intensity. At least five fields were evaluated (20x magnification) for each dam. Two researchers blinded to the experimental groups performed independent evaluation. An average score was then calculated.

### 2.4 qPCR

Mouse yolk sac samples (n = 8/group) were placed in RNA Later solution (Invitrogen, MA, USA) and kept at −20 °C until total RNA extraction, which was performed using Trizol (Invitrogen). Concentration and purity of total RNA were assessed by spectrophotometric analysis (Implen nanofotometer, Munich, Germany). Total RNA (1 μg) reverse transcribed into cDNA was undertaken using the High Capacity cDNA Reverse Transcription kit (Applied Byosistems, CA, USA), according to the manufacturer's instructions. A primer-containing mix (intron-exon spanning) of each gene of interest (Table 1), was incubated with cDNA (4 u L), and with the fluorescent DNA interlayer Eva Green (Biotium, CA, USA), in a total volume of 6 μL. Expression of each gene of interest was quantified relative to the geometric mean of the reference genes, *Ywhaz, Ppib* and *Gapdh* (Table 1). Expression analyses were undertaken in groups exhibiting stable expression of these reference genes. Analyses were performed using the QuantStudio Real-Time PCR system (Applied Biosystems, CA, USA), following these specific amplification cycles: initial denaturation at 50 °C for 2 min and then at 95 °C for 10 min, followed by 40 cycles of denaturation at 95 °C for 15 s. Annealing was performed at 60 °C for 30 s and extension at 72 °C for 45 s. Changes in mRNA gene expression were calculated using the 2-^ΔΔCT^ method [26] with acceptable efficiency ranging from 95 % to 105 %.

**Table 1:** Primers used for qPCR.

Primer	Sequence (5′- 3′)	Encoded Protein	Reference
Abca1	F:GCAGATCAAGCATCCCAACT R:CCAGAGAATGTTTCATTGTCCA	Abca1	[38]
Abcb1a	F:CTCTATTGGACAAGTGCTCACTG R:CTCCTCGTGCATTGGCGAA	P-glycoprotein (P-gp)	[38]
Abcb1b	F:AAGCCAGTATTCTGCCAAGCAT R:CTCCAGACTGCTGTTGCTGATG	P-glycoprotein (P-gp)	[38]
Abcg1	F:GCTCCATCGTCTGTACCATCC R:ACGCATTGTCCTTGACTTAGG	Abcg1	*
Abcg2	F:GCCGTTAGGACGCTCGCAGA R:TAGCAACGAAGACTTGCCTCCGC	Breast cancer related protein (Bcrp)	[39]
Snat1	F:GGACGGAGATAAAGGCACTC R:CAGAGGGATGCTGATCAAGG	Snat1	[46]
Glut1	F:CCAGCTGGGAATCGTCGT R:CAAGTCTGCATTGCCCATGAT	Glut1	[40]
Ccl2	F:AGGTCCCTGTCATGCTTCTG R:ATCTGGACCCATTCCTTCTTG	C-C Motif Chemokine Ligand 2 (Ccl2)	[41]
IL-6	F:GAGGATACCACTCCCAACAGACC R:AAGTGCATCATCGTTGTTCATACA	Interleukin (Il)-6	[42]
IL-1β	F:TTGACGGACCCCAAAAGATG R:AGAAGGTGCTCATGTCCTCA	Interleukin (Il)-1β	*
Mif	F:GCCAGAGGGGTTTCTGTCG R:GTTCGTGCCGCTAAAAGTCA	Macrophage inhibitory factor (Mif)	[47]
Cxcl1	F:ACCCGCTCGCTTCTCTGT R:AAGGGAGCTTCAGGGTCAAG	C-X-C Motif Chemokine Ligand 1 (Cxcl1)	[42]
Ywhaz	F:GAAAAGTTCTTGATCCCCAATGC R:TGTGACTGGTCCACAATTCCTT	Ywhaz	*
Gapdh	F:TGTGTCCGTCGTGGATCTGA R:TTGCTGTTGAAGTCGCAGGAG	Gapdh	[43]
Ppib	F:GAGACTTCACCAGGGG R:CTGTCTGTCTTGGTCCTCTCC	Ppib	*

*Gene specific primers were designed with primer-BLAST (http://www.ncbi.nlm.gov/tools/primer-blast).

### 2.5 Statistical method

Data are displayed as mean ± standard error of the mean (SEM) and were generated using Prisma program (GraphPad Software Inc., CA, USA). Statistical analysis of volumetric proportions, immunohistochemistry and qPCR data was conducted in the yolk sac of fetuses presenting the closest placental weight to the mean weight of all placentae from each litter. Therefore, “n” represents the number of litters [10,27,44] Non-parametric distribution of the data was tested whereas search of outliers was conducted using Grubbs' test. Differences between groups were assessed using non-parametric Mann-Whitney test to compare two variables, whilst Kruskal Wallis test, followed by Dunn's post-test was used to compare more than two variables. Statistical significance was considered when p < 0.05.

## 3 Results

### 3.1 Lipopolysaccharide (LPS) does not alter the volumetric proportion of the yolk sac’s histological components

**Fig. 1:**
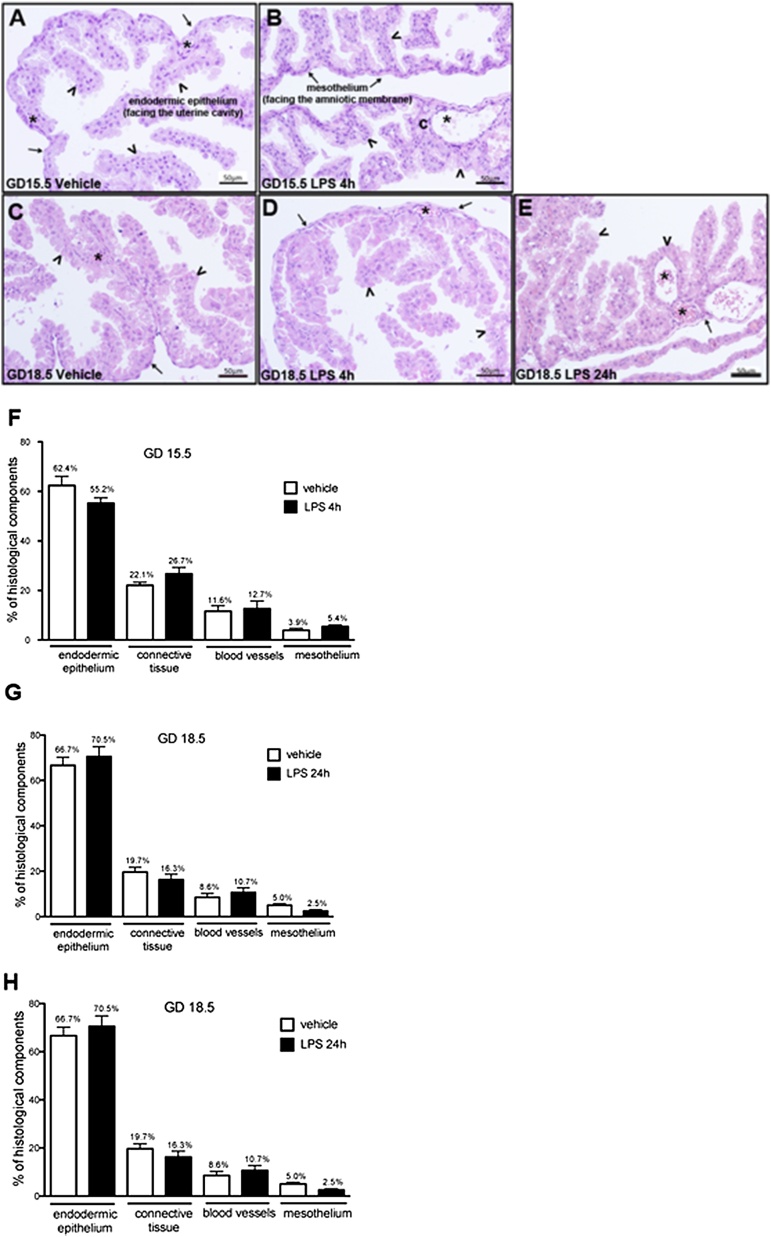
Histology and morphometric evaluation of the murine yolk sac following LPS exposure (n = 8/group). A and B: photomicrographs of histological sections of the yolk sac from mice at GD15.5 after 4 h of treatment with vehicle (A) and LPS (B). C, D and E: photomicrographs of histological sections of the yolk sac from mice at GD18.5 after 24 h of treatment with vehicle (C), and after 4 h (D) and 24 h (E) of treatment with LPS. Arrowheads: endodermic epithelium; arrows: mesothelium; asterisks: mesodermic blood vessels. Hematoxylin-eosin.F, G and H: Volumetric proportion of the histological components of yolk sac from mice at GD15.5 after 4 h of LPS treatment (F) and from mice at GD18.5 after 4 h (G) and 24 h (H) of LPS treatment. Kruskal Wallis test, followed by Dun's post-test. Statistical significance was considered when p < 0.05.

Analysis of H&E-stained sections by optical microscopy showed that the visceral yolk sac of animals from all experimental groups exhibited typical morphology, i.e., formed by three typical layers: the outer (uterine facing) endodermic epithelium, the middle mesenchymal tissue and the inner (amnion facing) mesothelium (Fig. 1A-E). Upon quantification, there were no significant changes in the volumetric proportions of the histological elements of the visceral yolk sac at GD15.5 4 h and at GD18.5, 4 or 24 h, after LPS treatments, compared to the proportions observed in controls (Fig. 1F–H). Of importance, change in tissue cellularity indicative of widespread agglomeration of mono and / or polymorphonuclear nuclei cells (a frank indicator of inflammation) was not observed.

### 3.2 Increase of yolk sac Abcg2 expression following lipopolysaccharide (LPS) exposure

**Fig. 2:**
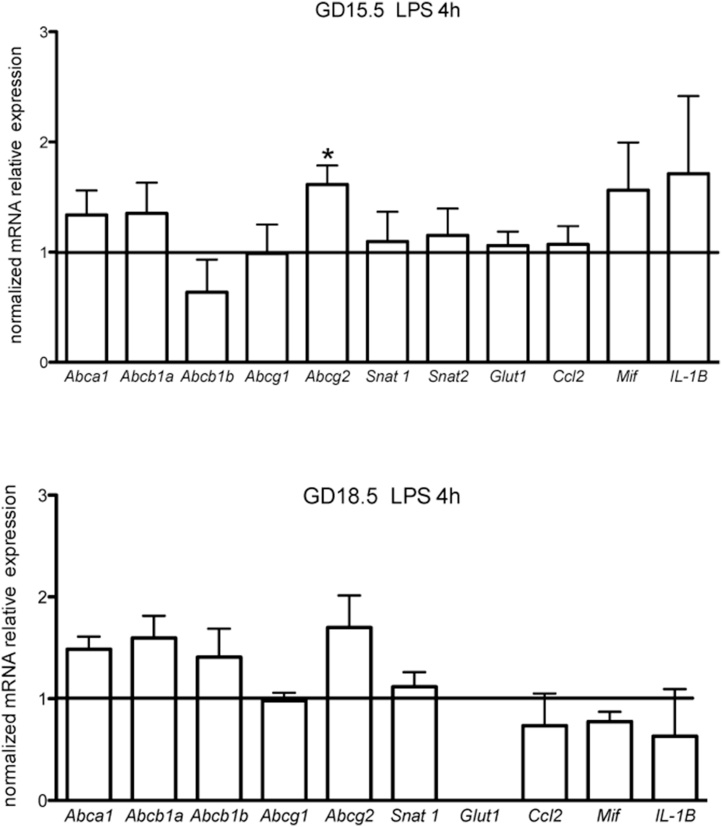
Relative mRNA expression of selected ABC transporters, nutrient transporters and cytokines in the yolk sac from LPS-treated mice (n = 8/group). *p < 0,05, Mann Whitney test.

The ABC transporter genes, *Abca1*, *Abcb1a*, *Abcb1b*, *Abcg1* and *Abcg2*, were expressed in the yolk sac at GD15.5 and GD18.5. However, their relative levels were only assessed in the 4 h groups at both gestational ages and not in the 24 h LPS group at GD18.5. The latter group did not exhibit steady expression levels of all reference genes evaluated, compared to control (data not shown), preventing us from undertaking relative expression analysis. Yolk sac *Abcg2* mRNA was upregulated at GD15.5 4 h following LPS treatment, (p < 0.05). But it remained unchanged at GD18.5 (4 h). No changes in *Abca1*, *Abcb1a*, *Abcb1b*, and *Abcg1* were observed regardless of the gestational age (Fig. 2). We also investigated the expression levels of two influx transport systems in our model, the Snat1 *(Slc38a1)* amino acid and the Glut1 *(Slc2a1)* glucose transporters. Yolk sac *Snat1* mRNA was detected at both gestational ages, whist *Glut1* was only detected at GD15.5 (4 h). LPS did not alter the mRNA expression of *Snat1* and *Glut1* at any time point investigated (Fig. 2).

Interleukin (*Il)-1β,* Macrophage migration inhibitory factor *(Mif)* and C–C motif chemokine ligand 2 (*Ccl2)* were expressed in the yolk sac at both gestational ages, but were unaffected by LPS (Fig. 2). Whereas, *Il-6* and the chemokine (C-X-C motif) ligand 1(*Cxcl1)* transcripts showed very low/undetectable levels of expression at both gestational ages, exposed or not to LPS (data not shown).

### 3.3 Yolk sac Bcrp is gestational-age dependently modulated by lipopolysaccharide (LPS)

**Fig. 3:**
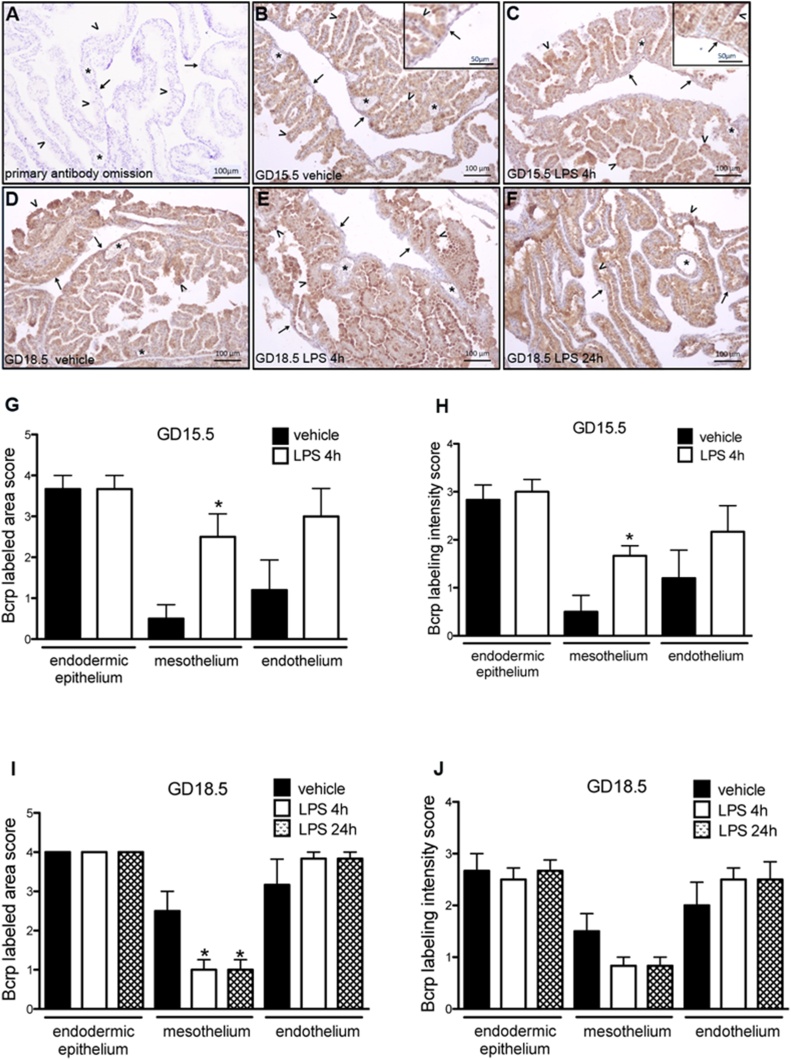
Immunohistochemistry for Bcrp protein in the yolk sac from LPS treated mice (n = 8/group). A: negative control of technique by primary antibody omission. B and C: Bcrp immunolabeling in the yolk sac from mice at GD15.5 after 4 h of treatment with vehicle (B) and with LPS (C). D, E and F: Bcrp immunolabeling in the yolk sac from mice at GD18.5, after 24 h of treatment with vehicle (D), and after 4 h (E) and 24 h (F) of treatment with LPS. Arrowheads: endodermic epithelium; thin arrows: mesothelium; asterisk: mesodermal blood vessels. Note the stronger intensity of labeling in the mesothelium of animals at GD15.5 after 4 h of LPS treatment (insert in C), when compared to those from animals at the same age treated with vehicle (insert in B). G, H, I and J: Semiquantitative evaluation of area and intensity of Bcrp immunolabeling. At GD15.5 both area (G) and intensity (H) of immunolabeling augmented after 4 h of LPS treatment. At GD18.5 it was observed a reduction in the area (I) of immunolabeling after 4 h and 24 h of LPS treatment. *p < 0,05, Mann Whitney test.

Bcrp immunolocalization was detected in the three yolk sac layers in vehicle and LPS-treated animals across pregnancy. Specifically, Bcrp was localized in the endodermal epithelium layer, with its apical surface being more intensely stained. The mesothelium also exhibited Bcrp immunostaining, whereas in the mesodermal layer, immunostaining was restricted to the endothelium of blood vessels, with the connective tissue displaying no labeling (Fig. 3B–F). Increased intensity and area of Bcrp immunostaing were detected in the mesothelium 4 h after LPS exposure at GD15.5. Conversely, decreased area of mesothelial Bcrp immunolabeling was detected in both time points at GD18.5 (p < 0.05), though changes in intensity of immunolabeling Bcrp did not attain significance (Fig. 3G-J).

**Fig. 4:**
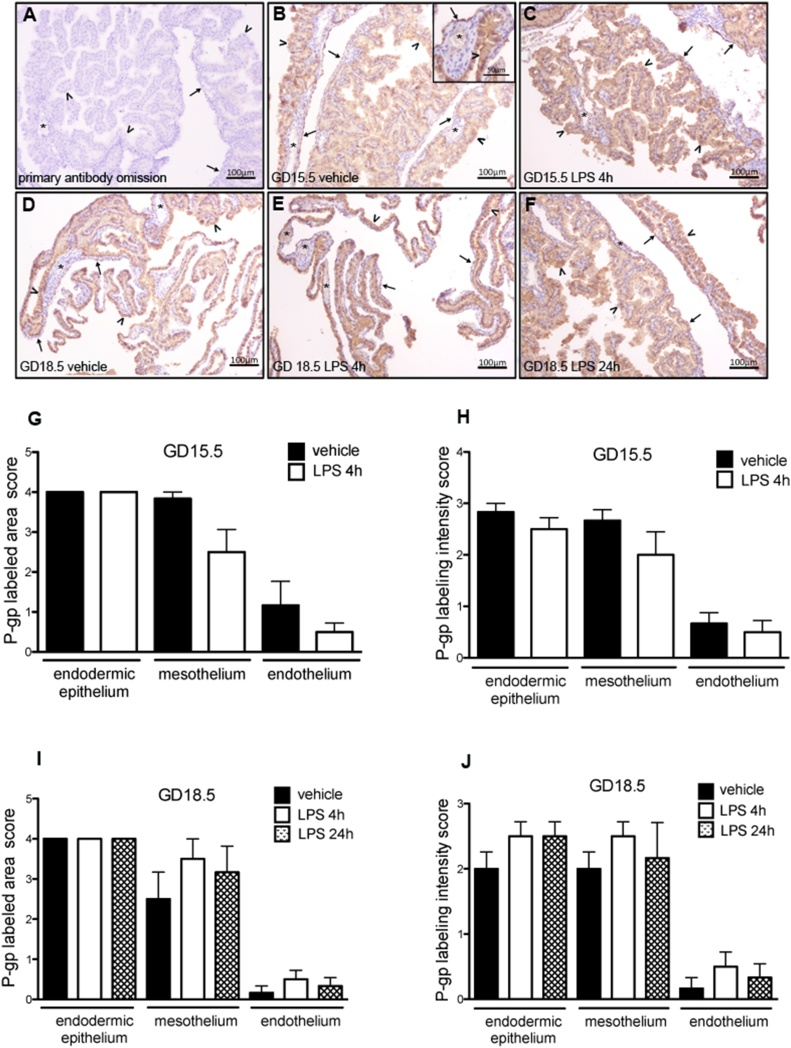
Immunohistochemistry for P-gp protein in the yolk sac from LPS treated mice (n = 8/group). A: negative control of technique by primary antibody omission. B and C: P-gp immunolabeling in the yolk sac from mice at GD15.5 after 4 h of treatment with vehicle (B) and with LPS (C). In the insert in B, it is possible to observe the endodermic epithelium and an area of the mesothelium intensely stained, while the endothelium of a blood vessel displays no labeling. D, E and F: P-gp immunolabeling in the yolk sac from mice at GD18.5, after 24 h of treatment with vehicle (D), and after 4 h (E) and 24 h (F) of treatment with LPS. Arrowheads: endodermic epithelium; thin arrows: mesothelium; asterisk: mesodermal blood vessels. G, H, I and J: Semiquantitative evaluation of area and intensity of P-gp immunolabeling. No significant differences were observed. *p < 0,05, Mann Whitney test.

P-gp was expressed in the yolk sac of animals from all experimental groups (Fig. 4B–F). P-gp immunostaining was more evident in the apical membranes, but it could also be observed in the cytoplasm of endodermal cells and mesothelial cells. In the mesothelium, variable P-gp immunolabeling was observed, with some areas being more intensely immunostained (Fig. 4B–F). The endothelium of the blood vessels showed little or no labeling, while the connective tissue of the mesodermal layer was completely negative (Fig. 4B, insert). There were no differences in the area or intensity of P-gp labeling, when comparing LPS-treated to control groups (Fig. 4G–J).

**Fig. 5:**
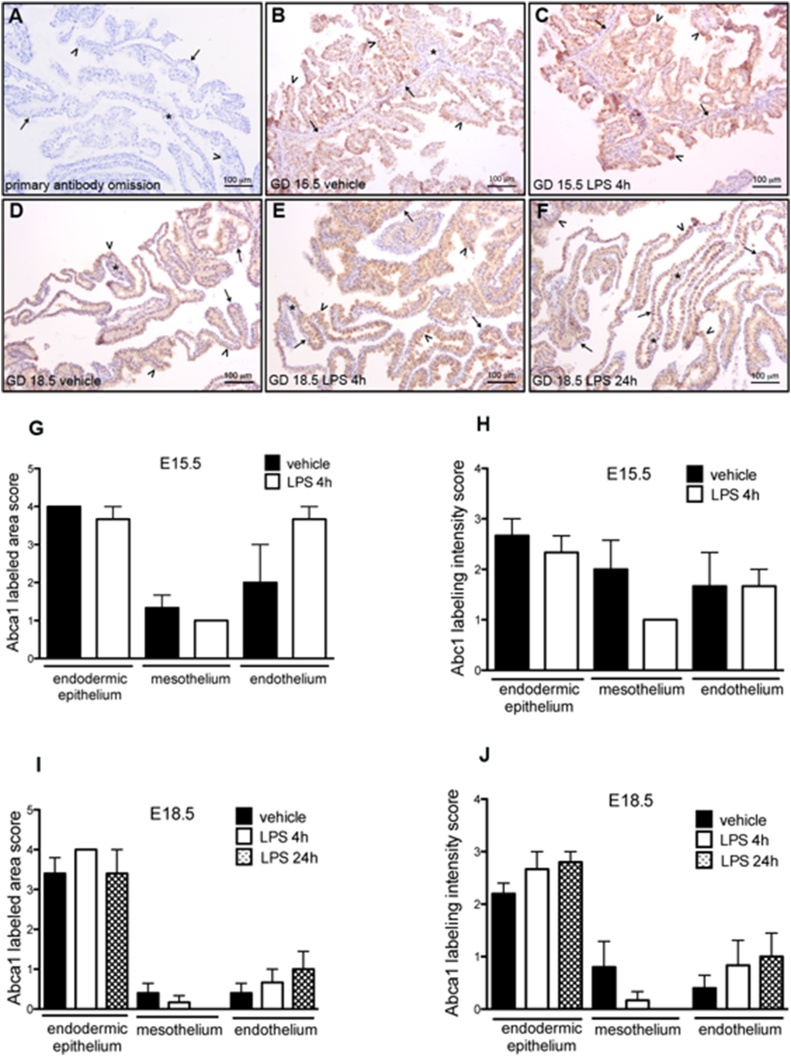
Immunohistochemistry for Abca1 protein in the yolk sac from LPS treated mice (n = 8/group). A: negative control of technique by primary antibody omission. B and C: Abca1 immunolabeling in the yolk sac from mice at GD15.5 after 4 h of treatment with vehicle (B) and with LPS (C). D, E and F: Abca1 immunolabeling in the yolk sac from mice at GD18.5, after 24 h of treatment with vehicle (D), and after 4 h (E) and 24 h (F) of treatment with LPS. Arrowheads: endodermic epithelium; thin arrows: mesothelium; asterisk: mesodermal blood vessels. G, H, I and J: Semiquantitative evaluation of area and intensity of P-gp immunolabeling. No significant differences were observed. *p < 0,05, Mann Whitney test.

The lipid efflux transporter Abca1 was primarily immunolocalized to the endodermal epithelium in all experimental groups (Fig. 5B–F). Heterogeneous Abca1 labeling was observed along the endodermal epithelium. The mesothelium and endothelium of the mesodermal vessels were also positive for Abca1, however the area of labeling was more variable, with labeled and unlabeled areas. The connective tissue of the mesodermal layer was immunonegative (Fig. 5B–F). There were no significant differences in the area or intensity of labeling for Abca1, when comparing the yolk sac of animals treated with LPS with those treated with vehicle, in the two gestational ages evaluated (Fig. 5G–J).

## 4 Discussion

This study shows that bacterial infection in pregnancy has the potential to alter the yolk sac barrier permeability to drugs and toxins through gestational-age dependent disruption of Bcrp/*Abcg2*. This disruption appears to occur without concomitant alterations in yolk sac morphology or a discernible local pro-inflammatory response.

Previously, we have demonstrated that malaria in pregnancy (MiP), an important inducer of preterm labor, upregulated term yolk sac P-gp and Abca1 expression [23]. These previous results, together with those presented herein, indicate that different infective agents potentially disrupt yolk sac efflux transport potential via alterations in the expression of selected ABC efflux transport systems, which may alter the biodistribution, within the embryo/fetal compartment, of endogenous and exogenous substrates present in the extraembyonic coelom, in the uterine cavity and / or in the yolk sac circulation. Whether these changes, in response to infection, are detrimental or beneficial to the developing conceptus remains to be determined.

In the pharmacological and toxicological context, results obtained at GD15.5 suggest that LPS-induced upregulation of Bcrp in the amnion facing-mesothelium, favors the entry of Bcrp substrates into the embryo/fetal compartment. In this context, Bcrp substrates of clinical importance comprise **1) xenobiotics:** including antibiotics, antiretrovirals, sulfonylureas, nonsteroidal anti-inflammatory drugs (NSAIDs), proton pump inhibitors (PPIs), and **2) toxicants**: including select mercuric species, estrogenic mycotoxins, carcinogens phototoxic compounds [19,[28], [29], [30], [31]]. We have previously demonstrated a decrease in placental Bcrp expression in mice exposed to LPS in the same experimental design and dose regimens [32]. As such, it is possible that LPS exposure decreases placental Bcrp and increases yolk-sac mesothelium Bcrp levels, resulting in increased accumulation of xenobiotics and toxicants in the fetal circulation and amniotic cavity. These changes may increase the likelihood of adverse pregnancy outcomes, including miscarriage, intrauterine growth restriction, preterm/early labor and / or fetal demise. In later stages of pregnancy (GD18.5), we detected a lower area of Bcrp staining in the mesothelium-yolk sac, showing a gestational-age dependent effect of LPS on yolk sac Bcrp expression.

Accordingly, in humans, expression of placental ABC transporters has been consistently demonstrated to be modulated by infection. LPS treatment of first trimester human placental explants and HTR8/SVneo (human extravillous trophoblast-like) cells, inhibited the expression BCRP/*ABCG2* with concomitant increase in mRNA levels of *IL-6*, *IL-8* and *CCL2* [32,33]. Conversely, BCRP/*ABCG2* levels were found elevated in human preterm placental fragments from patients with chorioamnionitis [20,22]. These divergent results from cell culture and *ex-vivo* experiments were probably a result of the considerable heterogeneity of human chorioamnionitis when compared to the TLR4-targeted effect of LPS [8,22]. Together, the data obtained in mice and humans show that LPS is capable of altering the expression of BCRP/*Abcg2* regardless of the gestational organ, ie. the yolk sac or placenta. This may therefore, modulate the transit of different substrates at the fetal-maternal interface. ABC transporters are highly conserved and display similar functions in mammalian species [34], thus it is possible that BCRP modulation by LPS has the potential to negatively impact pregnancy outcome across mammal species. Furthermore, the results presented herein also open of the possibility that the expression of other ABC efflux transporters (e.g., Abcc1-6, Mrp1-6) not investigated herein may be altered with LPS infection.

In the present study, sub-lethal LPS injections did not elicit a pro-inflammatory response in the yolk sac. Previously, we have shown a marked maternal and placental pro-inflammatory response to intraperitoneal injection of LPS [32]. Our data suggest that the placenta is capable of limiting the maternal pro-inflammatory transfer into the yolk sac, or alternatively the yolk sac is less capable of mounting a robust pro-inflammatory response to LPS infection. This is consistent with our previous findings that MiP induced a massive maternal and placental pro-inflammatory response, without activating a substantial yolk sac cytokine/chemokine output [10,23]. In this context, it is possible that other routes of LPS injection could have elicited a stronger pro-inflammatory response, since inoculation of LPS (1 mg/kg) directly into the amniotic cavity for 6 h induced fetal membrane upregulation of *Il-1β*, and *Il-6* at GD15/16 in DBA/2 mice [35]. Of importance, in this study, the authors did not dissect the yolk sac from the amnion membrane and used a higher dose of LPS and a different duration of treatment. These differences in experimental design may underlay the different results between the studies.

Previous studies have shown that the expression of two important influx nutrient transporters in the placenta, Snat1 (*Slc38a1*) amino acid and the Glut1 (*Slc2a1*) are altered by LPS [14,23,27,36,37]. In the present study, we did not observe differences in their expression in the yolk sac mRNA, likely due to absence of local pro-inflammatory response in the yolk sac.

One limitation of our study was not being able to evaluate gene expression after 24 h of LPS exposure at GD18.5. We evaluated the expression of 6 candidate reference genes in the yolk sac from control and LPS treated groups and all exhibited changes in expression levels between groups. This prevented us from normalizing the mRNA expression of our genes of interest. This difference in reference gene profile was likely due to the vicinity of labor, since C57BL/6 mice undergo labor from GD18 to 22 [10] and thus reflects the molecular and structural changes prior to fetal membrane rupture during labor.

In conclusion, we have demonstrated for the first time, that the yolk sac protective barrier modulated by Bcrp/*Abcg2* is disrupted by bacterial infection in a gestational-age dependent manner. The nature of this disruption appears different from that in the placenta. Higher expression of mesothelium-yolk sac Bcrp during bacterial infection may result in increased exposure of the embryo/fetus to an array of xenobiotics and toxicants. A number of these factors are known to have long-term health consequences in postnatal life.

## Declaration of Competing Interest

The authors report no declarations of interest.
